# Impact of Succinylcholine vs. Rocuronium on Apnea Duration for Rapid Sequence Induction: A Prospective Cohort Study

**DOI:** 10.3389/fmed.2022.717477

**Published:** 2022-02-09

**Authors:** Lijun Tang, Xiao Zhao, Shitong Li, Lina Huang, Jinbao Li, Lianhua Chen, Shiwei Huang

**Affiliations:** ^1^Department of Anesthesiology, Shanghai General Hospital of Nanjing Medical University, Shanghai, China; ^2^Department of Anesthesiology, Shanghai General Hospital, Shanghai Jiao Tong University, Shanghai, China

**Keywords:** rapid sequence induction intubation, succinylcholine, rocuronium, apnea duration, oxygen saturation

## Abstract

**Objective:**

The present study aimed to evaluate the impact of 1.5 mg/kg succinylcholine or 1.2 mg/kg rocuronium, vs. 1.0 mg/kg succinylcholine on apnea duration in patients underwent rapid sequence induction (RSI).

**Methods:**

This prospective cohort study was conducted in the Department of Anesthesiology in Shanghai General Hospital from July 2020 to November 2020. Apnea duration was defined as the time from apnea prompted by the P_ET_CO_2_ waveform to the time the point of oxygen saturation declined to 90% (T90) and 95% (T95) after succinylcholine or rocuronium administration. The primary outcome included T90 and T95 changes in 1.5 mg/kg vs. 1.0 mg/kg succinylcholine groups and 1.5 mg/kg succinylcholine vs. 1.2 mg/kg rocuronium groups.

**Results:**

A total of 265 participants were subjected for analysis. The succinylcholine (1.0 mg/kg) group had a significantly longer T90 (50.72, 95% confidence interval [CI, 7.60, 94.38], *P* = 0.015) and T95 (48.09, 95% CI [7.11, 89.07], *P* = 0.012) than the succinylcholine (1.5 mg/kg) group. In addition, significantly longer T90 (56.84, 95% CI [16.24, 97.44], *P* = 0.003) and T95 (50.57, 95% CI [12.58, 88.57], *P* = 0.003) were observed in the rocuronium (1.2 mg/kg) group than those in the succinylcholine (1.5 mg/kg) group. No severe side events were observed during the operation.

**Conclusion:**

Rocuronium and the lower dose of succinylcholine may be recommended to patients underwent RSI.

## Introduction

Rapid sequence induction (RSI) is a special technique of anesthetic induction designed to reduce the risk of aspiration of secretions of any kind, e.g., regurgitated or vomited gastric contents. This is done by shortening the normal sequence of intubation procedures and omitting certain steps to minimize the time between loss of consciousness and swallowing reflexes until the airway is secured by tracheal intubation (1). In particular, RSI involves not using mask ventilation, at least for adults.

Neuromuscular blockade agents (NMBAs) are used to facilitate endotracheal intubation (2). Succinylcholine is a short-acting depolarizing NMBA that is the most commonly conventionally used NMBA in RSI because of its fast onset and short duration (3). Unfortunately, it can have serious side effects (4, 5). Rocuronium, a steroidal non-depolarizing NMBA, is an alternative to succinylcholine due to its fast onset (6, 7). However, the 1.0–1.2 mg/kg dose recommended for RSI has too long a duration of action that fatal hypoxia is imminent if the airway is difficult, especially if intubation and mask ventilation fail simultaneously. This disadvantage of rocuronium is not eliminated by the very rapid reversal with sugammadex (8), because this drug is often not available fast enough in emergency situations (9). Therefore, the selection of succinylcholine or rocuronium in RSI should be carefully considered and tailored to the specifics of each patient and their clinical indications and medical conditions.

Apnea caused from not using mask ventilation, however, includes the risk of hypoxemia. Therefore, “non-hypoxic apnea,” defined as duration of apnea with SpO2 > 90%, is relevant to patient safety (10). Previous studies focused mainly on increasing oxygen storage to prolong non-hypoxic apnea duration (10, 11), whereas few studies have been performed on approaches to decrease oxygen consumption. Two aspects should be considered in the assessment of the optimal dose of succinylcholine and rocuronium in RSI: prolonging the non-hypoxic apnea duration (without additional oxygen consumption) and shortening the apnea interval (fast onset and good intubation conditions). Succinylcholine may lead to increased dose-dependent oxygen consumption (12). Our previous study showed that in obese patients the non-hypoxic apnea duration in the treatment with 1.5 mg/kg succinylcholine was shorter than that in the treatment with 0.9 mg/kg rocuronium (13), we assumed that fasciculation may be a potential cause of the shorter non-hypoxic apnea. However, poor intubation conditions and repeated intubation may also increase oxygen consumption. Succinylcholine is superior to rocuronium in terms of muscle relaxation effects in both good intubation conditions and clinically acceptable intubation conditions at quantities of 2–3 times the ED95 dose (6). Similar onset times and intubation conditions were achieved at doses higher than 3 times the ED95 dose (1.0 mg/kg−1.5 mg/kg succinylcholine and 1.0 mg/kg−1.2 mg/kg rocuronium) (7, 14–16). Therefore, the optimal doses of succinylcholine and rocuronium for application in RSI are still inconclusive.

Therefore, the aim of the present study was to compare the non-hypoxic apnea duration of the administration of 1.5 mg/kg succinylcholine, 1.0 mg/kg succinylcholine, and 1.2 mg/kg rocuronium during RSI.

## Materials and Methods

### Study Design

This prospective cohort study was conducted in the Department of Anesthesiology in Shanghai General Hospital from July 2020 to November 2020. This study was approved by the Ethics Committee of Shanghai General Hospital ([2020]54) and was registered in the Chinese Clinical Trial Registration Center (http://www.chictr.org.cn/index.aspx) with the registration number ChiCTR2000034769. Written informed consent was obtained from all individual participant.

### Participants

Patients who underwent elective surgery requiring RSI were enrolled. The following inclusion criteria were used: (1) American Society of Anesthesiologists (ASA) physical status I or II and (2) Age between 18 and 65 years. The exclusion criteria were as follows: (1) Unwilling to provide written informed consent; (2) Pregnancy; (3) Patients with a history of difficulty in intubation or failed intubation; (4) Obstructive sleep apnea syndrome; (5) A history of respiratory tract infection within a month; (6) Smoking cessation <2 months before surgery; (7) History of alcohol or drug abuse; (8) Cerebrovascular disease and increased intracranial pressure; (9) Drugs antagonists of rocuronium (such as phenytoin, carbamazepine, or protease inhibitors). In case one of the following conditions was present, the observation was to be suspended and the participant withdrawn from the study: (1) After preoxygenation, the end-tidal oxygen concentration was <90%; (2) Coughing during tracheal intubation; (3) Failed tracheal intubation twice times or more; (4) Recovery from spontaneous breathing before mechanical ventilation; (5) Severe allergic reactions; (6) Patients with circulatory fluctuations that were difficult to correct after 5 min of using vasoactive drugs. The participants were not compensated for their study participation.

### Grouping and Masking

General anesthesia drugs and treatment strategies were selected based on clinical needs. The choice of a muscle relaxant was jointly decided by the anesthetist and the participants. As an observational exploratory study, no blindness was applied.

### Typical Procedures

All subjects fasted for more than 8 h before surgery. After entering the operation room, a 20G indwelling vein cannula was placed in the median vein of the left antecubital fossa, and 10 mL/kg of sodium lactate Ringer's solution was infused to substitute for the fasting-induced fluid deficit. Then, 2% local lidocaine anesthesia was administered for the puncture and the placement of the left radial artery. Next, a pressure sensor was connected to the patient to measure the direct arterial pressure and perform blood gas analysis. If the left radial artery puncture and placement failed, the right forearm was used to measure the non-invasive blood pressure with a measurement interval of 1 min. Further, the subjects were connected to a GE CARESCAPE Monitor B650 (GE Healthcare Finland OY, Helsinki, Finland) to monitor the heart rate (HR), mean arterial pressure (MAP), finger pulse oxygen saturation (SpO_2_), bispectral index (BIS) and nasopharyngeal temperature (Temp). The subject lied in the supine position and was kept warm using a medical insulation blanket. The oxygen flow rate used was 10 L/min; the oxygen concentration reached was 100%. The airway pressure-limiting valve of the anesthesia machine was fully opened, the breathing circuit was pre-filled, and the subject was instructed to breathe calmly under a closed mask for 3 min. Then, an intravenous injection of fentanyl 3 μg/kg and propofol 2 mg/kg was administered. After the eyelash reflex disappeared and the subject ceased respond to the patting call, the muscle relaxant was administered (following the decision previously made by the anesthetist and the participants). Before the spontaneous breathing disappeared, the subject was to fasten the mask to avoid inhaling air; the mandible had to be unsupported before intubation, and any manual or mechanical ventilation was not to be applied. Fifty seconds after the administration of the muscle relaxant, the mask was removed, and a video laryngoscope (UETDC-K3) was utilized to perform a laryngoscopy to expose the glottis. Next, 60 s after the administration of the muscle relaxant, endotracheal intubation was conducted (7.5-mm cuffed tracheal tube for men and 7-mm cuffed tracheal tube for women). The insertion depth of the tracheal tube was 1 cm after the cuff was fully inserted into the glottis. After the tracheal intubation, a fiberoptic bronchoscope (UESCOPETIC-I1) was used to check whether the tracheal tube position is correct. The tracheal tube was opened in the air, without mechanical ventilation, and the end-expiratory carbon dioxide output was observed to monitor the recovery of spontaneous breathing. After tracheal intubation, to prevent the recovery of spontaneous breathing, intravenous bolus of rocuronium (0.6 mg/kg) was given to maintain muscle relaxation in the succinylcholine (1.5 mg/kg) and succinylcholine bromide (1.0 mg/kg) groups. To prevent consciousness restoration during anesthesia, propofol was intravenously injected at a rate of 5 mg /kg/h (Willi's Ark CONCERT-III infusion pump). In case BIS was >60, intravenous injection of 20 mg of propofol was applied. When SpO_2_ dropped to 90%, the tracheal tube was immediately connected to the anesthesia machine (GE Carestation 620) for mechanically controlled mechanical ventilation (parameter settings: oxygen concentration 100%, oxygen flow 1 L/min, tidal volume 8 mL/kg, frequency 16 beats/min, inhale-to-exhale ratio 1:2).

During the anesthesia, the hemodynamic parameters were monitored in real time. If HR > 110 beats/min, intravenous bolus of 1 mg of esmolol was administrated; if HR <50 beats/min, an intravenous bolus injection of atropine 0.5 mg was given. If SBP > 170 mmHg, intravenous bolus injection of 1 mg perdipine was administered, and if SBP <80 mmHg or MAP dropped more than 25% of the baseline value, 50 μg oxypinephrine was applied as an intravenous bolus injection.

### Outcomes

The primary outcome was the non-hypoxic apnea duration. That is, the time interval between P_ET_CO_2_ waveform area prompts apnea to oxygen saturation declined to 95% (T95) and 90% (T90) of 1.5 mg/kg succinylcholine vs. 1.0 mg/kg succinylcholine groups, and 1.5 mg/kg succinylcholine vs. 1.2 mg/kg rocuronium groups.

Prespecified secondary outcomes included the T95 and T90 of the 1.0 mg/kg succinylcholine and 1.2 mg/kg rocuronium groups and adverse events. The exploratory outcomes included the exploration of factors that correlated with T90, and the variation of blood gas analysis, BIS, temperature, HR, MAP, SpO_2_, ETO_2_, and P_ET_CO_2_.

Any adverse reactions, such as laryngospasm, bronchospasm, and masseter spasm, or muscle rigidity during intubation, were recorded during the surgery operation. Any adverse events found were followed up before improvement or discharge.

### Data Collection and Definition

The data collected included gender, age, ASA classification, preoperative hemoglobin (Hb), hematocrit (Hct), weight, height, body-mass index (BMI, kg/m^2^), smoking history, Mallampati score, T95, T90, duration of muscle fibrillation (TF, the time from the onset of muscle fibrillation to the disappearance of muscle fibrillation after intravenous injection of muscle relaxants), classification of the degree of muscle fibrillation, and the conditions of tracheal intubation. In addition, the laryngoscope exposure classification, number of intubation attempts, HR, MAP, SpO_2_, Temp, BIS, ETO_2_, as well as for at room entry, 3 min after oxygen inhalation, 30 s after muscle relaxant administration, 50 s after muscle relaxant administration, 2 min after intubation, SpO_2_ reduction to 95%, SpO_2_ decrease to 90%, SpO_2_ increase to 96%. Moreover, blood gas analysis was performed of pH, pO_2_, pCO_2_, cLac at room entry, 3 min after oxygen inhalation, and SpO2 decrease to 90%.

Muscle fibrillation was classified using the scale scores described in a previous report (17): 0 = No muscle tremor; 1 = Mild: slight muscle tremor in eyes, neck, face, or fingers, no limb movement; 2 = Moderate: moderate muscle tremor or obvious limb movement on both sides; 3 = Severe: severe or continuous and extensive muscle fibrillation. Endotracheal intubation condition was evaluated by Copenhagen score (18): 1 = excellent; 2 = good, and 3 = poor. Laryngoscope exposure classification (Cormack-Lehane classification) was defined as follows: 1 = the glottis was mostly visible; 2 = only the posterior union of the glottis could be seen, but not the glottis; 3 = only the epiglottis was visible; 4 = no glottal epiglottis could be seen. We used the World Health Organization classification for BMI: <18.5 (underweight), 18.5–24.9 (normal range), 25–29.9 (overweight), and >30 (obesity).

### Sample Size Calculation

Preliminary analysis revealed that T90 in the succinylcholine (1.5 mg/kg), succinylcholine (1.0 mg/kg), and rocuronium (1.2 mg/kg) groups was 475.9 ± 64.7, 534.7 ± 64.7, and 528.7 ± 52.5, respectively. Therefore, an estimated sample size of 91 patients per group could provide 80% power to detect a between-group difference, assuming a two-sided significance level of 2.5%. Considering a loss of follow-up of 10%, a number of 101 subjects per group are required.

### Statistical Analysis

Continuous variables that follow normal distribution was expressed with mean ± Standard deviation (SD), otherwise presented as median and interquartile range (IQR). Categorical variables were displayed using number and percentages. For multiple demographic characteristics comparison, nonparametric test was applied for continuous variables that not conformed to normal distribution. The comparison between the 1.5 mg/kg succinylcholine vs. 1.0 mg/kg succinylcholine and 1.5 mg/kg succinylcholine vs. 1.2 mg/kg rocuronium groups were analyzed using student *t* test. Two-way repeated measurement ANOVA was used to detect the effects of treatment and time interaction on HR and MAP. In addition, categorical variable comparison was conducted using Chi-square or Fisher exact tests. Pearson correlation analysis was employed to assess the correlation between T90 and the interested variables. All data were analyzed using SPSS 22.0 (IBM Corp., Armonk, NY, USA). *P* < 0.05 was considered statistically significant.

### Patient and Public Involvement

Not applicable.

## Results

**Figure 1:**
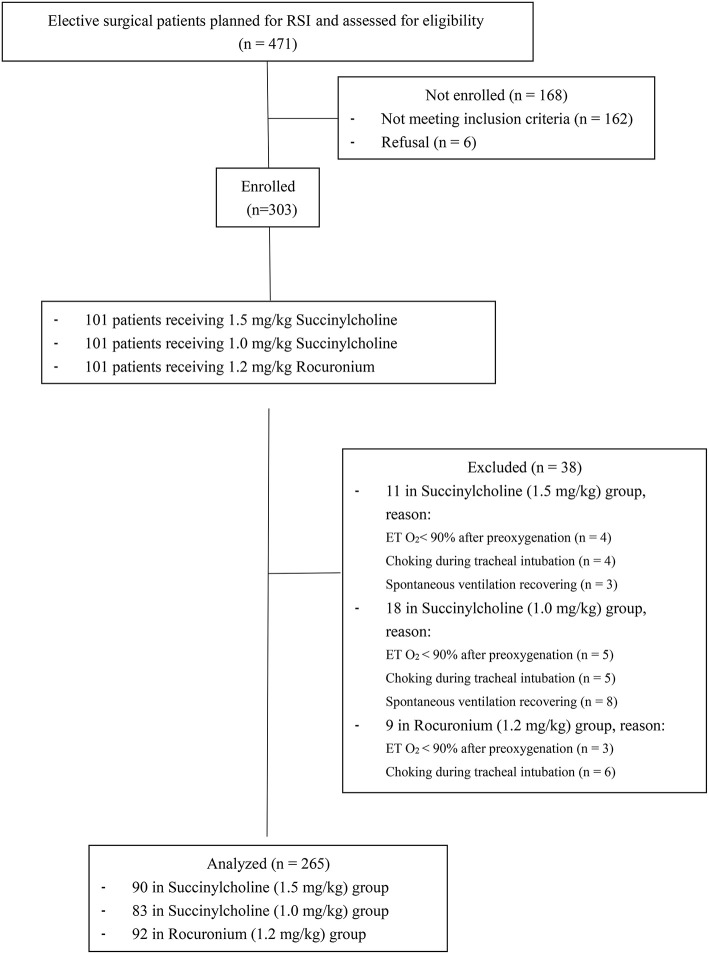
Flow chart.

A total of 471 patients were assessed for eligibility, of which 162 failed to meet the inclusion criteria, and 6 refused to participate. Of the enrolled 303 patients, 38 were excluded because of incomplete data for T90 and T95. Therefore, a total number of 265 subjects were analyzed: 90 cases in the 1.5 mg/kg succinylcholine, 83 in the 1.0 mg/kg succinylcholine, and 92 in the 1.2 mg/kg rocuronium groups (Figure 1). The demographic data are presented in Table 1.

**Table 1:** Clinical characteristics.

	**Succinylcholine**	**Rocuronium**	**Succinylcholine**
	**(1.5 mg/kg)**	**(1.2 mg/kg)**	**(1.0 mg/kg)**
	**(*n* = 90)**	**(*n* = 92)**	**(*n* = 83)**
Age, median (IQR)	39 (32, 52)	41 (32, 51)	43 (33, 52)
Gender			
Male	31 (34.4%)	43 (46.7%)	24 (28.9%)
Female	59 (65.6%)	49 (53.3%)	59 (71.1%)
Hemoglobin (g/L)	135 ± 13	137 ± 13	134 ± 11
Hematocrit	0.41 ± 0.04	0.41 ± 0.04	0.41 ± 0.03
Body weight (kg), median (IQR)	60 (54, 69)	65 (55, 72)	61 (54, 67)
Height (m)	1.63 ± 0.08	1.66 ± 0.09	1.63 ± 0.08
BMI (kg/m^2^)	23.3 ± 3.0	23.4 ± 3.0	23.0 ± 2.8
<18.5	3 (3.3%)	3 (3.2%)	3 (3.61%)
18.5–24.9	65 (72.2%)	61 (66.3%)	62 (74.7%)
25–29.9	21 (23.3%)	27 (29.4%)	16 (19.3%)
≥30.0	1 (1.1%)	1 (1.1%)	2 (2.4%)
ASA			
1	70 (77.8%)	69 (75.0%)	63 (75.9%)
2	20 (22.2%)	23 (25.0%)	20 (24.1%)
Mallampati			
1	25 (27.8%)	27 (28.3%)	24 (28.9%)
2	47 (52.2%)	52 (56.5%)	46 (55.4%)
3	18 (20.0%)	13 (14.1%)	13 (15.7%)
Smoking No	87 (96.7%)	82 (89.1%)	70 (84.3%)
Yes	3 (3.3%)	10 (10.9%)	13 (15.7%)
Medical history None	70 (77.8%)	69 (75%)	63 (75.9%)
High blood pressure	11 (12.2%)	8 (8.7%)	6 (7.2%)
Diabetic mellitus	4 (4.4%)	5 (5.4%)	8 (9.6%)
Hypothyroidism	3 (23.1%)	6 (6.5%)	4 (4.8%)
Hepatitis B	2 (2.2%)	4 (4.3%)	2 (3.0%)

*BMI, body-mass index; IQR, interquartile range*.

T90 in the 1.2 mg/kg rocuronium group was significantly longer (56.84 [95% CI 16.24–97.44], *P* = 0.003) than that in the 1.5 mg/kg succinylcholine group. Additionally, T90 in the 1.0 mg/kg succinylcholine group was statistically significantly longer (50.72 [95% CI 7.06–94.38], *P* = 0.015) than that in the 1.5 mg/kg succinylcholine group. Compared with the 1.5 mg/kg succinylcholine group, the 1.2 mg/kg rocuronium group had a significant longer T95 (50.57 [95% CI 12.58–88.57], *P* = 0.003). When compared with the succinylcholine (1.5 mg/kg) group, the succinylcholine (1.0 mg/kg) exhibited a significant longer T90 (48.09 [95% CI 7.11–89.07], *P* = 0.012).

For secondary outcome comparison, the T90 (6.12 [95% CI −34.58–46.82], *P* = 0.48) and T95 (2.48 [95% CI 35.74–40.70], *P* = 0.54) values were comparable between the 1.0 mg/kg succinylcholine and 1.2 mg/kg rocuronium groups (Table 2). No severe adverse events were observed during the surgery and during follow-up in all groups. A total number of 23, 13, and 19 mild adverse reactions occurred in the 1.5 mg/kg succinylcholine, 1.2 mg/kg rocuronium, and 1.0 mg/kg succinylcholine groups, respectively. Among them, sore throat and myalgia were the most common side effects (Table 3).

**Table 2:** Non-hypoxic apnea duration.

	**Succinylcholine (1.5 mg/kg)**	**Rocuronium (1.2 mg/kg)**	**Succinylcholine (1.0 mg/kg)**	
	**(*n* = 90)**	**(*n* = 92)**	**(*n* = 83)**	
T90 (s)	404 (310, 511)	487 (385, 578)	446 (385, 551)	
T95 (s)	354 (267, 450)	420 (333, 513)	392 (337, 493)	
**Primary outcome**				
	**Rocuronium vs. Succinylcholine**		**Succinylcholine (1.0 mg/kg)**	
	**(1.5 mg/kg)**	**P**	**vs. Succinylcholine (1.5 mg/kg)**	**P**
T90 Change (95% CI)	56.84 s (16.24, 97.44)	0.003	50.72 s (7.06, 94.38)	0.015
T95 Change (95% CI)	50.57 s (12.58, 88.57)	0.003	48.09 s (7.11, 89.07)	0.012
**Secondery outcome**				
	**Rocuronium vs. Succinylcholine**			
	**(1.0 mg/kg)**	**P**		
T90 Change (95% CI)	6.12 s (−34.58, 46.82)	0.48		
T95 Change (95% CI)	2.48 s (−35.74, 40.70)	0.54		

*T90, oxygen saturation declined to 90%; T95, oxygen saturation declined to 95%. CI, confidence interval*.

**Table 3:** Adverse events.

	**Succinylcholine**	**Rocuronium**	**Succinylcholine**
	**(1.5 mg/kg)**	**(1.2 mg/kg)**	**(1.0 mg/kg)**
	**(*n* = 90)**	**(*n* = 92)**	**(*n* = 83)**
Overall	23	13	19
Type of adverse events			
Sore throat	17	13	14
Myalgia	10	0	6
Degree of adverse events			
Mild	23	13	19
Severe	0	0	0

One incubation attempt was successfully performed in all patients. Tracheal intubation evaluation revealed that the incubation conditions, C-L classification, and incubation number were comparable among the groups. The time for muscle fibrillation was comparable between the 1.5 mg/kg and 1.0 mg/kg succinylcholine groups (*P* = 0.73) (Supplementary Table 1). The degrees of muscle fibrillation in these two groups were also similar (*P* = 0.11) (Supplementary Table 2).

**Figure 2:**
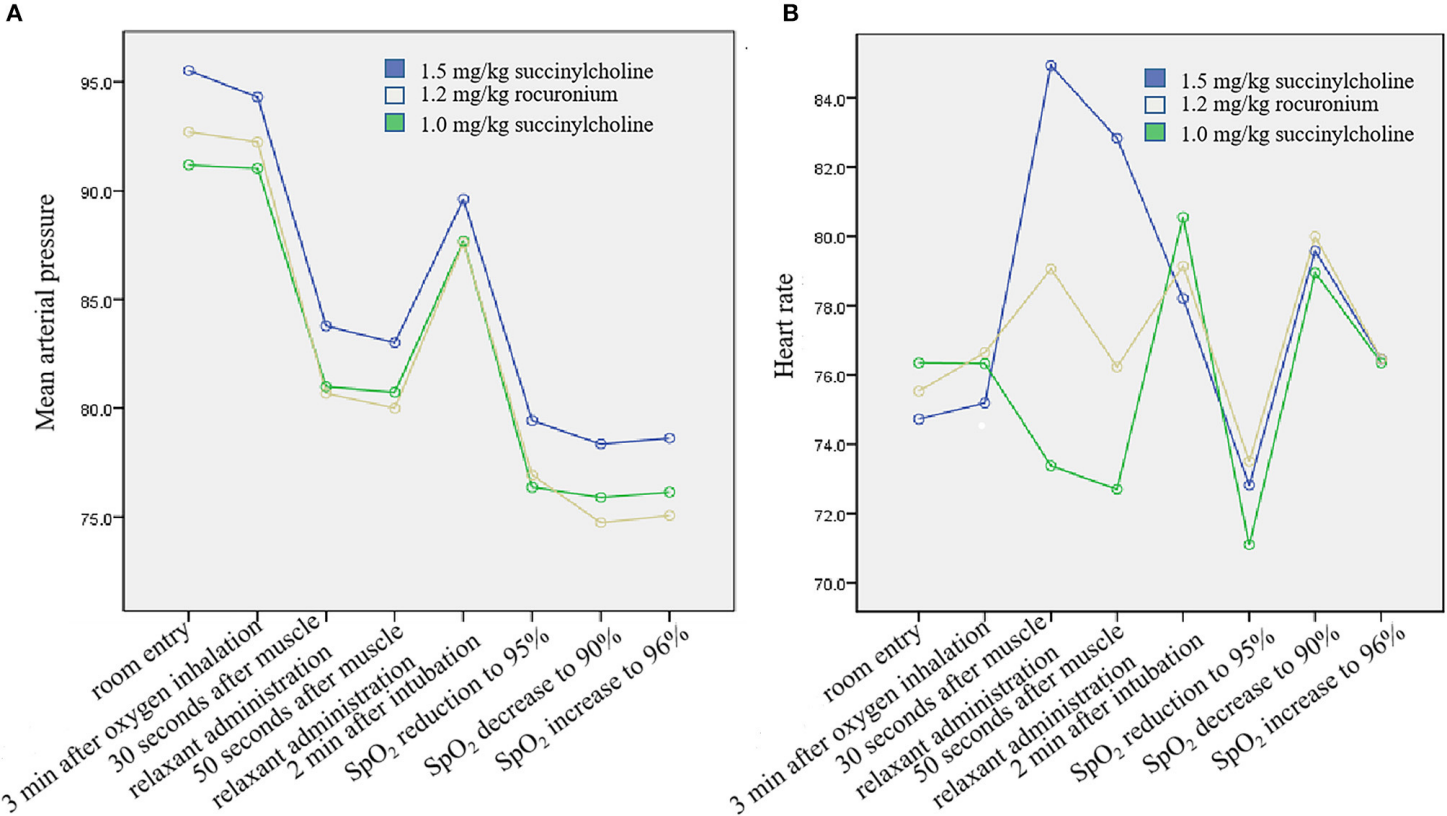
Mean arterial pressure and heart rate variation. **(A)** Mean arterial pressure. Two-way repeated measurement ANOVA suggested that time and treatment interaction was comparable (*P* = 0.279). **(B)** Heart rate. Two-way repeated measurement ANOVA suggested that time and treatment interaction showed statistical significance among the groups (*P* < 0.001), though no clinical significance was observed.

Moreover, the variation ratio of MAP and HR were within 30% at each time point (Figures 2A,B). In addition, the BIS variation among the three groups showed no clinical significance (Supplementary Table 3). The temperature during RSI was relatively stable, ranging from 36.0 °C to 37.1 °C.

Blood gas analysis showed a significantly different pH between room entry and 3 min after oxygen inhalation (*P* < 0.01) among the groups, despite no clinical significance. pO_2_ at 3 min after oxygen inhalation time point was highest in the 1.0 mg/kg succinylcholine group (*P* = 0.005). The pCO_2_ and cLac values significantly differed among the groups at room entry, 3 min after oxygen inhalation, and SpO_2_ decrease to 90% time points (all *P* < 0.01) (Table 4). The SpO_2_ in room entry and the minimum SpO_2_ time points were comparable among the groups (all *P* > 0.05). In addition, ETO_2_ was comparable among the groups, and P_ET_CO_2_ at 3 min after oxygen inhalation (*P* = 0.013) and SpO_2_ increase to 96% time points (*P* = 0.002) were significantly differed (Supplementary Table 4).

**Table 4:** Blood gas analysis.

	**Succinylcholine**	**Rocuronium**	**Succinylcholine**	**P**
	**(1.5 mg/kg)**	**(1.2 mg/kg)**	**(1.0 mg/kg)**	
	**(*n* = 90)**	**(*n* = 92)**	**(*n* = 83)**	
**pH**				
Room entry	7.40 (7.39, 7.42)	7.42 (7.40, 7.43)	7.41 (7.40, 7.43)	0.002
3 min after oxygen inhalation	7.40 (7.38, 7.42)	7.42 (7.40, 7.43)	7.41 (7.39, 7.42)	0.005
SpO_2_ decrease to 90%	7.30 (7.28, 7.33)	7.30 (7.28, 7.31)	7.31 (7.29, 7.32)	0.24
**pO**_**2**_ **(mmHg)**				
Room entry	86 (85, 88)	85 (82, 87)	86 (84, 87)	0.051
3 min after oxygen inhalation	457 (413, 493)	445 (401, 482)	479 (432, 517)	0.005
SpO_2_ decrease to 90%	63 (62, 65)	63 (62, 65)	63 (62, 65)	0.83
**pCO**_**2**_ **(mmHg)**				
Room entry	36.1 (34.7, 38.2)	37.4 (36.6, 38.5)	36.8 (35.4, 38.8)	0.003
3 min after oxygen inhalation	35.6 (32.7, 37.7)	39.15 (36.7, 41.6)	38.1 (35.7, 40.9)	<0.001
SpO_2_ decrease to 90%	52.8 (47.4, 57.7)	55.9 (51.4, 59.5)	57.2 (53.5, 62.4)	<0.001
**cLac (mmol/L)**				
Room entry	0.9 (0.8, 1.0)	1.0 (0.9, 1.2)	0.9 (0.7, 1.1)	<0.001
3 min after oxygen inhalation	0.9 (0.8, 1.1)	1.1 (1, 1.2)	0.9 (0.8, 1.1)	<0.001
SpO_2_ decrease to 90%	1.0 (0.9, 1.1)	1.1 (1, 1.2)	1.0 (0.8, 1.1)	<0.001

Correlation analysis was used to explore the potential factors that might affect T90. As depicted in Supplementary Table 5, BMI, age, and hemoglobin were significantly correlated with T90 (all *P* < 0.001). Gender, smoking, muscle fibrillation time period, and muscle fibrillation degree were not correlated with T90.

## Discussion

In the present study, compared with 1.5 mg/kg succinylcholine group, both 1.0 mg/kg succinylcholine and 1.2 mg/kg rocuronium groups had significantly longer T90 and T95. These findings may provide evidence that lower dose succinylcholine and 1.2 mg/kg rocuronium may be feasible in clinical practice.

RSI is a critical medical measure that facilitates intubation. The selection of neuromuscular relaxants has been extensively studied, but there is no consensus. One of the highest risks of RSI comes from hypoxemia and reducing hypoxemia risk should be considered in the choice of muscle relaxants. Nevertheless, the available evidence on non-hypoxic apnea duration is limited. A meta-analysis showed that succinylcholine was superior to rocuronium in achieving excellent clinically acceptable intubation conditions (6). However, no statistical difference in the intubation conditions achieved was found between rocuronium and succinylcholine (19). In terms of non-hypoxic apnea duration, it was suggested that 1.0 mg/kg succinylcholine and 1.2 mg/kg rocuronium were superior to 1.5 mg/ kg succinylcholine in T90 and T95. These findings suggested that the administration of 1.0 mg/kg succinylcholine may be safer than higher doses. We noted that apneic oxygenation during the apnea period in RSI did not prevent desaturation as compared with conventional care measures in cardiac or traumatic arrest patients (20).

In the present study, the T95 and T90 in the 1.0 mg/kg succinylcholine and 1.2 mg/kg rocuronium groups were significantly longer than that in the 1.5 mg/kg succinylcholine group. In a previous study, Taha et al. reported that the T95 value in 1.5 mg/kg rocuronium-treated patients was 378 (370–393), which was lower than that in the present study. This discrepancy may be attributed to the differences in the fentanyl dose, start time points, and sample sizes (21). Our previous study compared the time of oxygen saturation decline to 92% (T92) in 0.9 mg/kg rocuronium- and 1.5 mg/kg succinylcholine-treated obese patients, in which rocuronium showed a significantly longer T92 (13), which in agreement with the present study.

Succinylcholine can trigger muscle fibrillation and increase muscle fibrillation-related metabolism. In this study, the degree and duration in the 1.5 mg/kg succinylcholine and 1.0 mg/kg succinylcholine groups were comparable, which may be explained with the supply of oxygen by myoglobin, which minimized its effect on systemic oxygen reservation (22). Moreover, the heart rate was significantly accelerated in the 1.5 mg/kg succinylcholine group, whereas mild effect was observed in the 1.0 mg/kg succinylcholine and 1.2 mg/kg rocuronium groups. Previous study suggested that succinylcholine increased anaerobic metabolism and disturbed the tissue oxygen supply and demand balance, and that high-dose succinylcholine elevated the risk of hemoglobin desaturation (12). In animal experiments, continuous infusion of succinylcholine augmented oxygen consumption (23). These previous results together with our findings suggest that a high dose of succinylcholine during RSI may need to be avoided.

This study is not without limitations. Although there were no significant differences in the intubation conditions, due to the observational nature of the study, we could not conclude whether they showed comparable effects. Additionally, elder adults and obese patients, as well as such with lung-related diseases were not included, which limited the representativeness of the conclusion. We noted that gender and height significantly differed among the three groups, and whether these two parameters affect oxygenation desaturation needs further investigation. Moreover, patients failed to complete RSI, coughing during intubation, or spontaneous breathing before mechanical ventilation were excluded for analysis, which may introduce selection bias, so the interpretation of results should be cautious.

## Conclusion

In conclusion, this study revealed that a relatively low dose of succinylcholine and rocuronium led to a longer non-hypoxic apnea duration. Therefore, 1.0 mg/kg succinylcholine or 1.2 mg/kg rocuronium may be recommended for RSI to satisfy the required intubation conditions. Further large-scale randomized control studies are needed to validate these findings.

## Data Availability Statement

The original contributions presented in the study are included in the article/Supplementary Material, further inquiries can be directed to the corresponding authors.

## Ethics Statement

The studies involving human participants were reviewed and approved by Shanghai General Hospital. The patients/participants provided their written informed consent to participate in this study. Written informed consent was obtained from the individual(s) for the publication of any potentially identifiable images or data included in this article.

## Author Contributions

LT and XZ conceived and coordinated the study, designed, performed and analyzed the experiments, and wrote the paper. SL, LH, JL, LC, and SH carried out the data collection, data analysis, and revised the paper. All authors reviewed the results and approved the final version of the manuscript.

## Conflict of Interest

The authors declare that the research was conducted in the absence of any commercial or financial relationships that could be construed as a potential conflict of interest.

## Publisher's Note

All claims expressed in this article are solely those of the authors and do not necessarily represent those of their affiliated organizations, or those of the publisher, the editors and the reviewers. Any product that may be evaluated in this article, or claim that may be made by its manufacturer, is not guaranteed or endorsed by the publisher.
