# Supplementary material for: Impact of Succinylcholine vs. Rocuronium on Apnea Duration for Rapid Sequence Induction: A Prospective Cohort Study
Source: Front Med (Lausanne). 2022 Feb 9;9:717477. doi: 10.3389/fmed.2022.717477 (PMC8864070; doi:10.3389/fmed.2022.717477)
Supplement: Supplementary file 1 [file Table_1.docx]

**Supplementary Table 1. Incubation evaluation.**

|  | Succinylcholine (1.5 mg/kg)  (n = 90) | | Rocuronium (1.2 mg/kg) (n = 92) | Succinylcholine (1.0 mg/kg)  (n = 83) | *P* |
| --- | --- | --- | --- | --- | --- |
| Copenhagen score (reflecting intubation conditions) | | | | | 0.67 |
| Excellent | 80 (88.9%) | 79 (85.9%) | | 70 (84.3%) |  |
| Good | 10 (11.1%) | 13 (14.1%) | | 13 (15.7%) |  |
| Poor | 0 (0.0%) | 0 (0.0%) | | 0 (0.0%) |  |
| Cormack-Lehane grade |  |  | |  | 0.53 |
| 1 (best view) | 62 (68.9%) | 68 (73.9%) | | 61 (73.5%) |  |
| 2 | 26 (28.9%) | 19 (20.7%) | | 20 (24.1%) |  |
| 3 | 2 (2.2%) | 5 (5.4%) | | 2 (2.4%) |  |
| 4 (worst view) | 0 (0.0%) | 0 (0.0%) | | 0 (0.0%) |  |
| Number of intubation attempts | 1(100.0%) | 1(100.0%) | | 1(100.0%) | 1.00 |
