# Supplementary material for: Impact of Succinylcholine vs. Rocuronium on Apnea Duration for Rapid Sequence Induction: A Prospective Cohort Study
Source: Front Med (Lausanne). 2022 Feb 9;9:717477. doi: 10.3389/fmed.2022.717477 (PMC8864070; doi:10.3389/fmed.2022.717477)
Supplement: Supplementary file 2 [file Table_2.docx]

Supplementary Table 2. Muscle fibrillation.

|  | Succinylcholine (1.5 mg/kg) (n=90) | Succinylcholine (1.0 mg/kg)  (n=83) | *P* |
| --- | --- | --- | --- |
| The time of muscle fibrillation (s) | 54 (48, 62) | 54 (46, 63) | 0.73 |
| The degree of muscle fibrillation |  |  | 0.11 |
| 0 (none) | 9 (10.0%) | 12 (14.5%) |  |
| 1 (mild) | 45 (50.0%) | 50 (60.2%) |  |
| 2 (moderate) | 30 (33.3%) | 20 (24.1%) |  |
| 3(severe) | 6 (6.7%) | 1 (1.2%) |  |
