# Supplementary material for: Impact of Succinylcholine vs. Rocuronium on Apnea Duration for Rapid Sequence Induction: A Prospective Cohort Study
Source: Front Med (Lausanne). 2022 Feb 9;9:717477. doi: 10.3389/fmed.2022.717477 (PMC8864070; doi:10.3389/fmed.2022.717477)
Supplement: Supplementary file 3 [file Table_3.docx]

Supplementary Table 3. Bispectral indexes.

|  | Succinylcholine (1.5 mg/kg) (n=90) | Rocuronium (1.2 mg/kg) (n=92) | Succinylcholine (1.0 mg/kg) (n=83) | *P* |
| --- | --- | --- | --- | --- |
| room entry | 96 (95,97) | 95 (93,97) | 96 (95,98) | 0.005 |
| 3 min after oxygen inhalation | 95 (94,96) | 95 (93,97) | 95 (94,97) | 0.12 |
| 30 seconds after muscle relaxant administration | 43 (42,45) | 45 (42,46) | 43 (42,46) | 0.16 |
| 50 seconds after muscle relaxant administration | 42 (41,44) | 42 (41,44) | 43 (41,44) | 0.98 |
| 2 min after intubation | 43 (42,45) | 42 (41,44) | 43 (41,45) | 0.085 |
| SpO_2_ reduction to 95% | 42 (41,43) | 42 (41,43) | 42 (42,44) | 0.049 |
| SpO_2_ decrease to 90% | 42 (41,43) | 42 (41,44) | 42 (40,43) | 0.12 |
| SpO_2_ increase to 96% | 42 (41,43) | 42 (41,43) | 42 (40,43) | 0.94 |
