# Supplementary material for: Impact of Succinylcholine vs. Rocuronium on Apnea Duration for Rapid Sequence Induction: A Prospective Cohort Study
Source: Front Med (Lausanne). 2022 Feb 9;9:717477. doi: 10.3389/fmed.2022.717477 (PMC8864070; doi:10.3389/fmed.2022.717477)
Supplement: Supplementary file 4 [file Table_4.docx]

Supplementary Table 4. Values of SpO_2_, ETO_2_, and PETCO_2_.

|  | Succinylcholine (1.5 mg/kg) (n=90) | Rocuronium (1.2 mg/kg) (n=92) | Succinylcholine (1.0 mg/kg) (n=83) | *P* |
| --- | --- | --- | --- | --- |
| SpO_2_ (%) |  |  |  |  |
| room entry | 98 (97, 98) | 97 (97, 98) | 98 (97, 98) | 0.69 |
| Minimum | 88 (87, 88) | 88 (86, 89) | 88 (87, 89) | 0.23 |
| ETO_2_ (%) | 91 (90, 91) | 91(90, 92) | 91 (90, 92) | 0.10 |
| P_ET_CO_2_ (mmHg) |  |  |  |  |
| 3 min after oxygen inhalation | 33 (31, 35) | 34 (32, 34) | 34 (32, 35) | 0.013 |
| SpO_2_ decrease to 90% | 50 (45, 56) | 47 (45, 55) | 52 (46, 57) | 0.074 |
| SpO_2_ increase to 96% | 45 (39, 52) | 41 (38, 46) | 45 (40, 50) | 0.002 |
