# Supplementary material for: Impact of Succinylcholine vs. Rocuronium on Apnea Duration for Rapid Sequence Induction: A Prospective Cohort Study
Source: Front Med (Lausanne). 2022 Feb 9;9:717477. doi: 10.3389/fmed.2022.717477 (PMC8864070; doi:10.3389/fmed.2022.717477)
Supplement: Supplementary file 5 [file Table_5.docx]

Supplementary Table 5. Correlation analysis

|  |  | Age (year) | Muscle fibrillation degree | Muscle fibrillation time period | Gender | Smoking | Hb (g/l) | BMI (Kg/m2) |
| --- | --- | --- | --- | --- | --- | --- | --- | --- |
| T90 | r | -0.35 | -0.06 | -0.017 | 0.19 | -0.071 | -0.24 | -0.75 |
|  | p | <0.001 | 0.3 | 0.79 | 0.002 | 0.25 | <0.001 | <0.001 |

BMI, body-mass index; T90, P_ET_CO_2_ waveform to the time the point of oxygen saturation declined to 90%. Hb, hemoglobin.
